# Periphery and brain, innate and adaptive immunity in Parkinson’s disease

**DOI:** 10.1007/s00401-021-02268-5

**Published:** 2021-02-08

**Authors:** Ashley S. Harms, Sara A. Ferreira, Marina Romero-Ramos

**Affiliations:** 1 Department of Neurology and Center for Neurodegeneration and Experimental Therapeutics, The University of Alabama at Birmingham, Birmingham, AL USA; 2 Department of Biomedicine and CNS Disease Modelling Group, Aarhus University, Høegh-Guldbergsgade 10, 8000C Aarhus, Denmark

**Keywords:** Alpha-synuclein, Parkinson, Microglia, Monocyte, T-Cell, Neuroinflammation

## Abstract

Parkinson’s disease (PD) is a neurodegenerative disorder where alpha-synuclein plays a central role in the death and dysfunction of neurons, both, in central, as well as in the peripheral nervous system. Besides the neuronal events observed in patients, PD also includes a significant immune component. It is suggested that the PD-associated immune response will have consequences on neuronal health, thus opening immunomodulation as a potential therapeutic strategy in PD. The immune changes during the disease occur in the brain, involving microglia, but also in the periphery with changes in cells of the innate immune system, particularly monocytes, as well as those of adaptive immunity, such as T-cells. This realization arises from multiple patient studies, but also from data in animal models of the disease, providing strong evidence for innate and adaptive immune system crosstalk in the central nervous system and periphery in PD. Here we review the data showing that alpha-synuclein plays a crucial role in the activation of the innate and adaptive immune system. We will also describe the studies suggesting that inflammation in PD includes early changes in innate and adaptive immune cells that develop dynamically through time during disease, contributing to neuronal degeneration and symptomatology in patients. This novel finding has contributed to the definition of PD as a multisystem disease that should be approached in a more integratory manner rather than a brain-focused classical approach.

## Introduction

Parkinson’s disease (PD) is characterized by significant dopaminergic neuronal loss in the substantia nigra (SN) and intraneuronal aggregation of alpha-synuclein (α-syn) in Lewy bodies. During the last decade, research on the role of the immune system in PD has gained momentum. Multiple lines of evidence supporting the occurrence of a chronic inflammatory event have come forward from studies in human patients’ brain and biofluids (CSF and serum), as well as in animal models of PD. The research community is now proposing that the immune component in PD occurs early and changes dynamically with disease progression, contributing to neuronal degeneration and symptomatology in patients. In addition, research has shown that both brain, as well as peripheral immune cells, are involved in this inflammation where innate and adaptive immune systems are activated. This novel understanding has contributed to the definition of PD as a multisystem disease that should be approached in a more integratory manner rather than a brain-focused classical approach. In this review we will explore the most relevant research findings achieved during the last decade in regards to the immune system in PD, with a special focus on the role of α-syn.

### Microgliosis in Parkinson’s disease and early signs of neuroinflammation

The presence of microglia activation (i.e. increase in numbers and/or changes in morphology and protein expression) in the brain of PD patients has been shown by histopathological studies in *postmortem* tissue [74]. Multiple studies have reported an increase of cytokines in the brain and CSF, indicative of a pro-inflammatory profile and chronic inflammation in PD [102]. The initial proposed hypothesis suggested that microglia responded once neurons die, and that the subsequent immune activation is deleterious to the surviving neurons. Accordingly, numerous studies have supported the neurotoxic capacity of over-activated immune cells and pro-inflammatory cytokines (reviewed in [149]). Thus, it is likely that the neuronal death in PD is, at least partially, due to pro-inflammatory immune activation. However, whether this is a lack of anti-inflammatory ability (loss of function) or an acquired pro-inflammatory activity (gain of function) is yet unknown. How early in the disease this occurs has been a highly controversial factor in previous years and a focus of investigation by numerous labs. As a result, longitudinal characterization of PD models and extensive analysis of human PD brain tissue, have shown that microgliosis occurred prior to cell death or even in its absence [149]. Accordingly, microgliosis has been observed in *postmortem* brains from PD patients in areas where neuronal death is not significantly found [90], which is also confirmed by in vivo PET imaging [61, 132] using PK11195, a ligand of the peripheral benzodiazepine receptor/TSPO (upregulated on activated microglia and other immune-related cells). In addition, PD animal models have shown that the microglia response precedes the neuronal loss, further suggesting that the immune response occurs early, as neurons start degenerating, where neurotransmission and other functions are affected [149]. This is possible, because microglia express receptors that recognize proteins of neuronal origin, such as neurotransmitters. Moreover, it is known that the “homeostatic status of microglia” is actually an active state, achieved by the neuronal expression of molecules assertive of a “healthy” condition [11]. This might change when neuronal function is compromised in PD, consequently activating microglia prior to neuronal cell death. This early activation has been shown in multiple occasions in several PD models, both in rodents and in non-human primates (see below). Additionally, data obtained from REM sleep behavioural disorder (RBD) patients- considered high risk for developing PD and hence putative prodromal PD [14]- shows that microgliosis occurs years before a possible PD diagnosis [164]. Therefore, the microglia response occurs early in PD and might contribute to the disease progression. Whether the immune response is deleterious in each of the disease stages (prodromal, early and late) and whether it plays a role in the disease onset and aetiology is yet to be defined.

### α-synuclein as initiator of the immune response

Microglia activation is an early event in PD, as they sense early signs of neuronal dysfunction or distress and react accordingly. Among these signals, microglia can perceive changes in the structure of endogenous proteins like α-syn, where its fibrillation and antigenic development may initiate a sterile response by acting as a damage-associated molecular pattern (DAMP) (for a more detailed review we refer the reader to [51]). The inflammatory ability of α-syn was first suggested by seminal work from the Zhang lab [194] and the Federoff-Maguire-Zeiss’s team [165]. Such findings became especially relevant when the constitutive release of α-syn by neurons was described [109]. Moreover, it is speculated that the total amount of misfolded α-syn released by neurons will increase progressively through disease. This will have direct relevance regarding the immune response, as studies have shown the ability of α-syn to initiate a pro-inflammatory response is greater when the protein is misfolded (fibrils or oligomeric) [84] or influenced by PD-associated mutations [83, 144] and can be potentiated by extracellular vesicles derived from PD patients’ blood [68]. Significantly, the role of α-syn in activating innate immunity is true for both microglia and monocytic cells, as research has shown α-syn can activate myeloid cells in the brain and periphery [103, 111].

This proposed role of α-syn as DAMP has resulted in a plethora of works investigating the interaction of different forms of α-syn with a variety of membrane proteins expressed on microglia (and other myeloid cells) (Table 1). Of particular relevance, Toll-like receptors (TLR) 2 and 4, which are upregulated in monocytes and microglia in PD patients [40, 41, 97], have been proposed as key players in the innate immune process. Several labs have shown in vivo and in vitro that TLR2 recognizes oligomeric or fibrillar forms of α-syn, leading to pro-inflammatory signals, further resulting in neuronal degeneration and death [34, 72, 97]. Nonetheless, TLR4 seems to recognize all types of α-syn (monomeric and fibrillar) and while it initiates an inflammatory response, it also promotes α-syn clearance, supporting an alternative or protective role for TLR4 [50]. Accordingly, blocking TLR2 has been suggested to be neuroprotective, while activation or overexpression of TLR4 exerts protection in rodent models of α-synucleinopathies [98, 176].

**Table 1:** α-Syn binding receptors and consequent activated pathways

Receptors	α-Synuclein species	Pathways activated	References
*TLR2*	Oligomeric, fibrillar	p38 MAPK, NFκB/p65	[34, 72, 97]
*TLR4*	Monomeric, oligomeric, fibrillar	NFκB/p65	[50, 176]
*Integrin* α_M_β_2_; *CR3;*			
*CD11b (human)*	Monomeric, fibrillar	Phagolysosome formation (only fibrillar)	[93]
*CD11b (mouse)*	Oligomeric, fibrillar	NOX2; Chemotaxis	[96, 180]
*Integrin* α_X_β_2_; *CR4;*			
*CD11c (human)*	Fibrillar	Phagolysosome formation	[93]
*CD36*	Monomeric	NLRP3, ERK1/2	[165]
	Fibrillar	Fyn mediated α-syn uptake and NLRP3	[133]
*FcγR*	Oligomeric, fibrillar	NFκB/p65	[23]
*FcγR/IgG*	Oligomeric, fibrillar	Phagosome formation	[8, 71]

α-syn has been also shown to interact with complement receptor (CR)3 (integrin α_M_β_2_, or CD11b/CD18) and CR4 (integrin α_X_β_2_, or CD11c/CD18), which are known to mediate microglial phagocytosis. Aggregated α-syn interacts with CD11b leading to NOX2 activation and increased oxygen radicals in mice [87, 180]. Our recent study shows that, although human α-syn interacts with both human CR3 and CR4, the CR4 seems to have a specific role in recognition of the fibrillar forms. Moreover, conformational changes of the CRs were essential for the clearance of the protein and the phagolysosome formation [93], suggesting opposite roles of these membrane receptors. In addition, mouse integrins CD11b (α_M_β_2_) and β_1_ have been related to chemotaxis and microglia migration induced by α-syn [96, 180]. α-syn also interacts with CD36 [165] and together with fyn kinase (nonreceptor Src family tyrosine kinase), mediates α-syn uptake and subsequent NLRP3 inflammasome activation [133]. NLRP3 inflammasome activation has been shown to play an important role in the neurodegenerative process of different PD models, as targeting its activity is neuroprotective in rodent models [65, 120]. This is also supported by changes in IL-1β and NLRP3 in PD patients [29].

Therefore α-syn interaction with immune receptors is defined by the α-syn-type, contributes to its clearance and in parallel to immune activation. Thus, α-syn induces activation of intracellular cascades such as ERK and p38 MAPK, stimulation of the NFκB dependent gene transcription, and the NLRP3 inflammasome activation, all leading to induction of pro-inflammatory signals [38, 65, 72, 83, 97, 133]. Accordingly, ours and other’s studies have shown that extracellular α-syn leads to pro-inflammatory cytokine production and oxidative stress, ultimately resulting in neurodegeneration [65, 77, 78]. A cautionary note regarding the α-syn concentrations used in most studies, which are far from the physiological α-syn levels reported in human fluids [26] (for additional discussion see [51]). Moreover, paradoxically, CSF α-syn decreases in PD patients *vs.* healthy individuals [26], although oligomeric or misfolded seems to increase thus elevating antigenicity [76]. Similarly, in brain tissue, high molecular weight α-syn increases in synucleinopathies and the total protein is also increased in MSA, although not significantly in PD [173]. Therefore, α-syn immune activation could be related to local increased accumulation of misfolded antigenic α-syn in synapses, rather than a general increase of the protein.

### The role of microglia on the anatomical spreading of α-synuclein pathology

It is proposed that α-syn pathology can spread anatomically, and this might occur via release of misfolded α-syn and its subsequent uptake by an interconnected healthy cell, where misfolded α-syn can act as a template and promote aggregation. In this context, microglia may also play a central role in the disease progression via non-cell autonomous mechanisms. As mentioned, α-syn released by neurons activates microglia and this in turn might further promotes pathological α-syn modifications. For example, α-syn-induced inflammasome activation will trigger caspase-1 expression, which results in α-syn truncation [181]. Also, microglial activation promotes oxidative stress that can induce oxidation or nitration of α-syn [55, 160]. A recent study suggests that microglia produced exosomes are also involved in this spreading, as they can be a source of oligomeric α-syn and promoters of neuronal α-syn aggregation [39]. Thus, α-syn per se or other factors promoting microglia activation (as unrelated immune events, see below) will contribute to α-syn pathology, further perpetuating the disease.

Notably, microglia are the cells that best clear extracellular α-syn in the brain and this should prevent the spreading/seeding process [110]. However, when enhanced and uncontrolled, microglial clearance capacity might become toxic or impaired and contribute to synaptic degeneration. Indeed, LPS-induced microglia activation promoted host α-syn transfer to grafted neurons, corroborating the deleterious effect of overt pro-inflammatory activation. However, the depletion of microglia increased the transfer of α-syn among grafted neurons, supporting microglia relevance in α-syn clearance [60]. Controversially, another study suggests the opposite, since in their experimental design, microglia depletion decreased α-syn neuronal pathology after intracerebral injections of α-syn fibrils [39]. These discrepancies might exist due to the differential models used and more research is needed to understand the role of microglia in α-syn pathology and its spreading.

α-syn degradation in microglia involves autophagy and lysosomal clearance, which seems to be dependent on the activation profile of microglia, the type of α-syn (misfolded, mutated…), as well as the presence of other molecules. As mentioned, conformational changes of the CR are required for the α-syn related phagolysosome formation [93]. Some studies suggest increased phagocytosis upon activation with monomeric α-syn [135] while decreased if exposed to fibrillar α-syn [32, 190]. α-syn seems to induce lysosomal damage and oxidative stress [53]. Accordingly, a recent work suggests that fibrillar but not monomeric α-syn leads to lysosomal damage and, consequently, failure in autophagy in microglia, which results in mitochondria impairment and microglia cell death [21]. Despite this last aspect, the degeneration and death of microglia is poorly studied in PD.

**Fig. 1:**
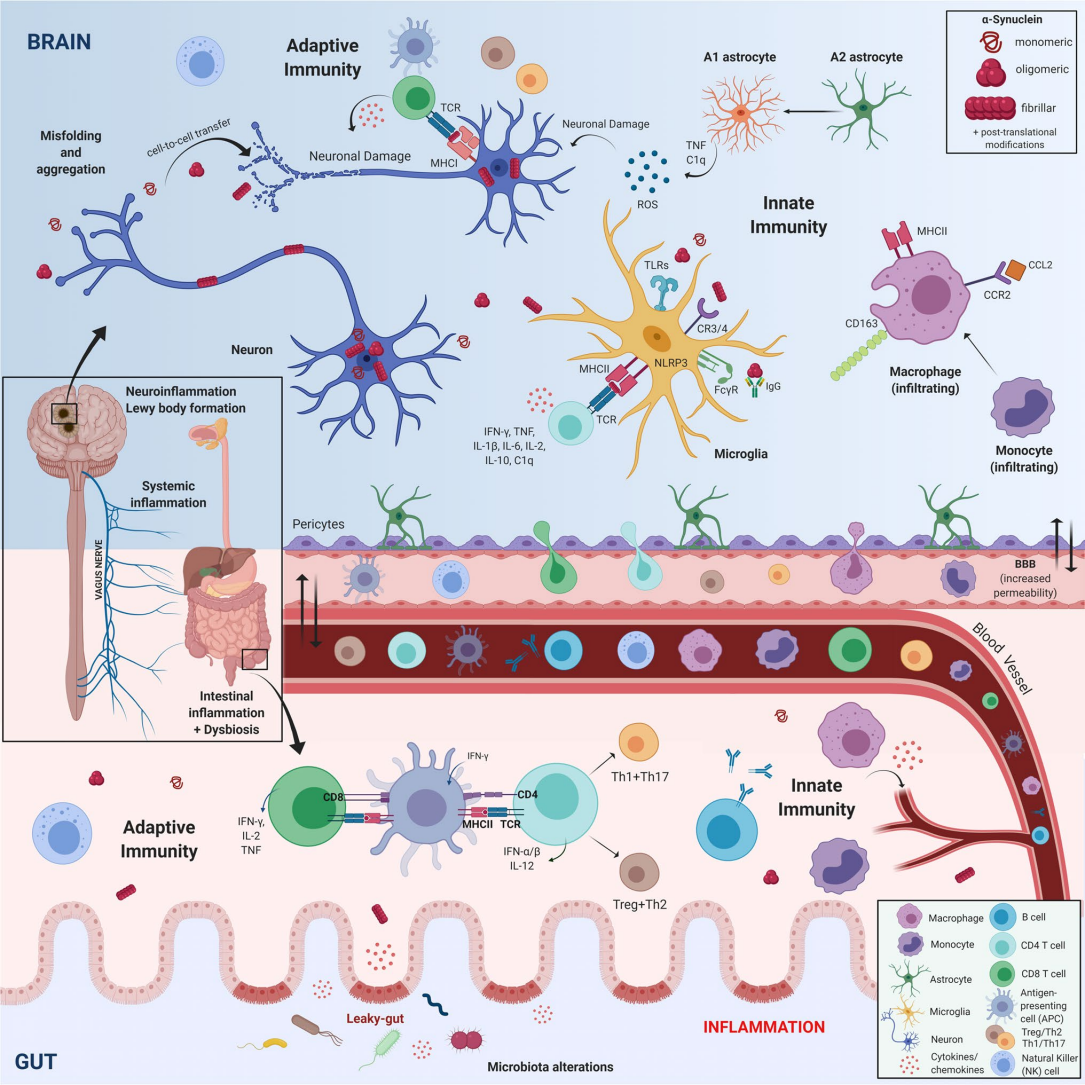
Immune response to alpha-synuclein induced neurodegeneration. During Parkinson’s disease, α-Synuclein (α-syn) undergoes post-translational modifications (phosphorylation, nitration, truncation…), forms oligomeric species and finally insoluble fibrils that aggregate in neurons in the Lewy Bodies. This process leads to neuronal dysfunction and ultimately cell death. Neurons can release monomeric or modified α-syn to the extracellular space. There, they should be cleared by microglia and/or infiltrating macrophages (CD163 + /CCR2 +), but also by astrocytes. If this process fails, the modified α-syn can be taken up by neighboring healthy neurons, where it will seed the aggregation of the endogenous α-syn. The modified α-syn will in parallel act as a damage-associated molecular pattern (DAMP) and via diverse immune receptors found in microglia and macrophages, induce a pro-inflammatory response. The pro-inflammatory cytokines will further promote neurodegeneration by direct action in neurons or indirectly by promoting A1 astrocyte differentiation. This innate immune response will also be accompanied by the adaptive immune response. The intracellular degradation of pathological α-syn will direct peptides of the protein to the MHC system that will in turn activate CD8 (T-cytotoxic cells, Tc), via MHCI, and CD4 (T-helper cells, Th), via MHCII. Such processes might occur both in the brain, but also in the periphery. Depending on the cytokines produced, the CD4-Th cells will undergo differentiation/maturation to Th1 or Th17 T-cells, which typically potentiate pro-inflammatory events; or into Th2 or Treg T-cells that will resolve the inflammation. In addition, B-cell activation will result in production of antibodies that will aid the clearance of α-syn and to NK activation, that can also help clearing α-syn. Additionally, this process will be influenced by parallel immune related events such as bacterial infections, intestinal inflammation, and changes of microbiota in the gut, which can increase gut permeability and result in a leaky gut-wall. This will change the immune milieu, possibly facilitating α-syn pathology and further promote inflammation. (Figure created using BioRender.com)

FcγRs have also been suggested to play a role on internalization and clearance of α-syn [23]. In that regard, the humoral response will be another factor affecting the clearance of α-syn, since the presence of antibodies increases efficiency of the FcγR-mediated clearance [8]. It is important to note that ageing, the major risk factor for PD, progressively decreases the ability of microglia (and macrophages) to phagocytose α-syn [12]. Under normal conditions in the CNS, microglia should be able to clear α-syn while supporting neuronal health through synaptic pruning and release of cytokines and growth factors. Notably in disease conditions, phagocytosis of pathological α-syn will lead the protein to the major histocompatibility complex (MHC) system and thus, the presentation of α-syn peptides by microglia (or other cells) to the adaptive system [77, 150]. As we will discuss in detail ahead, this leads to the recruitment of the adaptive immune system and to T-cell and B-cell activation (Fig. 1).

### Immune response in animal models of α-synuclein pathology

While it is currently debated whether neuroinflammation is an initiator, driver, or consequence of human PD, animal models have shown that inflammation precedes overt neurodegeneration, indicating that it is likely a driver of disease pathogenesis as interventional studies targeting the immune system are generally neuroprotective.

#### Immune response in the absence of neuronal death in α-synuclein transgenic models

To date, many animal models have been created that overexpress full-length human α-syn, genetic mutations or gene duplication events linked to familial PD (for review see [104]). These models have been instrumental in describing α-syn driven neuroinflammation and correlating synuclein burden with age and immune system dysfunction (reviewed in [91]). Thy-1-α-syn transgenic mice (line 61) have been extensively characterized for neuroinflammation as they show enhanced expression of TLRs 1,2,4, and 8 in the SN, as well as increased TNF mRNA at 6 months, MHCII expression on IBA-1 + cells at 14 months and increased CD4 and CD8 T cells in blood at 22 months of age [182], indicating that α-syn burden correlates with immune responses. Although, it should be noted that the transgene is driven by a Thy-1 promoter also highly expressed in immune cells thus complicating interpretation. The most recently generated BAC-α-syn rats show progressive α-syn aggregation and neurodegeneration, with an early microglia activation and important immune component [107]; that upon immunomodulation using resolvin D1, leads to neuroprotection. Autosomal dominant PD mutations in α-syn have also been incorporated into transgenic mouse models. While most of these models have not been characterized extensively for neuroinflammation or infiltration of peripheral immune cells, microgliosis (IBA-1 + cells) have been reported in association to α-syn pathology and neurodegeneration [48, 62, 64].

#### Innate and adaptive immunity are activated in the α-synuclein viral models

Viral-vector or overexpression models, particularly adeno-associated virus (AAV) models have long been popular and reliable for long-term, spatially restricted α-syn expression in neurons within the CNS. These vectors have been carefully designed to overexpress either human full length or familial mutations of α-syn in subsets of neurons in the CNS depending on serotype with low or non-existent transduction of glial support cells [177]. It should be mentioned that, while these models are great for studying local effects of α-syn overexpression in spatially restricted populations of neurons, once the neurons are transduced, there is no overt spreading or templating throughout the CNS as observed in human disease, making them poor models of α-syn transmission. Additionally, the use of viral vector models require careful validation and controls as the presence of viral entities may have neuroinflammatory effects on neurons and glia within the CNS.

Many serotypes have been used to transduce neurons in the basal ganglia to express human α-syn, those with the most extensive immune characterization being AAV2/1 [77, 171] and AAV2/5 [150]. Collectively, overexpression of α-syn in neurons within the SN in rodents results in robust expression of MHCII [77, 150] and CD68 [150, 171] on microglia and infiltration of CD4 and CD8 T cells [150]. Similarly, AAV2/5 mediated expression of human WT α-syn or A53T α-syn in non-human primates lead also to microglia proliferation, activation and increased MHCII expression, independently of the presence or absence of dopaminergic cell death; further confirming immune microgliosis as an early event during α-syn pathology [9]. Moreover, in this primate model we observed infiltration of B-cells (CD19 + HLA-DR-). Such B-cell infiltration was also seen upon AAV2/1 mediated α-syn expression in nigral neurons, which resulted as well in early pro-inflammatory cytokine expression (TNF, IL-6, iNOS), deposition of IgG within the ipsilateral midbrain [24, 171] and infiltration of CCR2 + monocytes prior to cell loss [80]. Importantly, showing the relevance of the MHCII-T cell interactions and the role of the adaptive system, genetic deletion of MHCII [77], or CIITA (transcriptional co-activator of MHCII) [186] attenuated α-syn mediated neuroinflammation and neurodegeneration (see below for further discussion). Similarly, the deletion of CCR2, essential for recruitment of the monocytic cells from periphery, also resulted in neuroprotection [80], which indicates that the peripheral component is crucial in driving the neurodegeneration observed in the AAV2/1 model.

#### The novel α-synuclein preformed fibril model: a yet undefined immune component

Mirroring the success of viral models and created to overcome the challenges of working with spatially restricted α-syn expression in neuronal subpopulations, many labs have transitioned to using α-syn preformed fibrils (PFF) [116, 178] or human Lewy body extracts to model templating and prion-like properties of α-syn in PD. When injected into the striatum of A53T α-syn transgenic mice (M83 line) [117], expansive propagation and templating of α-syn occurs throughout the CNS, modelling the “prion hypothesis” (reviewed in [118]). In rodents, α-syn propagation occurs along a connectome based upon region with the highest levels of endogenous α-syn. In non-transgenic mice, propagation is far more limited to interconnected brain regions leading to mild neurodegeneration over time, similar to what is observed in other viral and transgenic models with some detectable mild motor deficits [117].

The characterization of the early immune response in these models is underway and to date, studies focusing on neuroinflammation are limited. In mice, striatal injections of murine α-syn PFF activated the NLRP3 inflammasome in microglia, while the NLRP3-inhibitor MCC950 attenuated α-syn pathology, motor deficits, and neurodegeneration [65]. Further suggesting the deleterious microglia role, depletion of microglia in the PFF mouse model resulted in a partial decrease of the neuronal α-syn pathology [70]. One recent study has shown that the mouse model results in microgliosis, T-cell, natural killer (NK)-cell and B-cell infiltration, which was paralleled by changes in the peripheral immune populations in the spleen and lymph nodes [44]. In a follow up study, the team showed a protective role of infiltrating NK cells by scavenging α-syn and reducing pathology [45]. The model has also been associated with microglia activation that in turn promoted astrocyte conversion to an A1 neurotoxic phenotype, a process that, if avoided, resulted in neuroprotection [192].

Intranigral [78] or striatal injection [42] of mouse PFF α-syn fibrils into Sprague Dawley rats led to upregulation of MHCII and IBA-1 expression in the midbrain, accompanied by the infiltration of peripheral myeloid cells (CD163 +) and T cells prior to measurable neurodegeneration, indicating that innate and adaptive immune mechanisms are likely at play prior to overt neurodegeneration in the PFF models. Interestingly, the MHCII response is less robust in the mouse model (our own observations), which might be due to the interspecies difference in the rats when using murine (and not rat) PFF α-syn to trigger pathology. However more research is required to fully elucidate this. In that regard, certain factors should be considered for future studies in the PFF based model. First, the PFF concentrations used in the model (5–10 µg) are far from the α-syn physiological levels, thus the immune reaction observed in the area of injection might be an artefact. To overcome this, investigating the changes in neuronal and glia population of distant areas anatomically connected to the injection site and with α-syn pathology might more accurately reflect the immune response due to neuronal α-syn mishandling as it might occur in patients. Finally, the choice of control; if monomeric α-syn is used, this might reflect a “healthy response to α-syn” *vs.* the alternative control of PBS/saline corresponding to an absence of immune response.

### Peripheral immunity in Parkinson’s disease: evidence for monocyte involvement and infiltration

The presence of α-syn pathology in the periphery and the peripheral neuropathy in PD supports a more holistic affection of the nervous system in PD [31]. This peripheral α-syn pathology occurs early as it is also seen in RBD patients, prodromal PD [4]. Since the antigenicity of α-syn is true for both microglia and monocytes [103, 111] an innate immune response is expected to occur both in the brain and periphery, and their cross-talk will shape the integrated immune response in PD (Fig. 1). Accordingly we have shown that, in RBD patients, TLR4 expression in blood monocytes directly correlated to the immune brain activation and indirectly to the dopaminergic neurotransmission as shown by PET (Farmen, Romero-Ramos et al., unpublished), therefore supporting an early central and peripheral immune response, and a cross talk between brain and periphery that associates to the neurodegenerative event. However, it is unclear how much of this is exerted from the periphery or through recruitment and infiltration.

Infiltration of monocytes/macrophages in PD has been suggested based on the increased expression in the brain of proteins associated with non-microglia myeloid cells, such as CD163 and CCR2 (Fig. 1). We have shown an increase of the cells expressing the scavenger receptor CD163 in the brain of rodent PD models [78, 170], which is in agreement with findings in *postmortem* brains from PD patients [139]. Pharmacological anti-inflammatory modification of the CD163 + population in the periphery resulted in partial neuroprotection in the SN of the rat PD model [170]. The CCL2-CCR2 axis has been suggested to play an important role in the infiltration of monocytes into the inflamed brain [63]. We have shown that CCR2 + monocytes infiltrate the brain in an α-syn based PD mouse model and the genetic deletion of CCR2 was neuroprotective, suggesting a deleterious role for infiltrating monocytes in PD [80]. Further supporting a role for CCR2, the receptor was found upregulated in PD patients classical monocytes, even though the total number of CCR2 + monocytes was reduced [54], while another study showed activation of the CCR2-CCL2 axis [142] and CCL2 enrichment in patients’ blood [67]. Notably, differences in levels of CCL2 in serum or CSF seem to be associated to different clinical subtypes of PD [17, 73]. Therefore, CD163 + or CCR2 + monocytes seem to play a role in neurodegeneration in PD not only by their action in periphery, but also by infiltrating the brain.

#### Changes in monocyte subpopulations in Parkinson’s disease

Genetic profiling of monocytes can distinguish PD from healthy controls, supporting a disease associated response in this population [67]. This approach identified a distinct transcriptomic signature in monocytes from early PD patients, with differentially expressed genes such as HLA-DQB1 (MHCII system), MYD88 (associated to TLR2&4), REL (member of the NFκB transcription factors) and TNF [156], thus confirming the early relevance of the immune system in PD. Gene expression was also different in rapid *versus* slow PD progression phenotypes [140]. Although the data obtained in these studies is rather exploratory and at times overwhelming, they confirm the active involvement of monocytes early in the disease and their relevance in the clinical manifestation in patients.

Blood monocytes are usually subclassified based on CD14 and CD16 expression [95]. Classical monocytes (CD14 + /CD16-) show the highest expression of chemokine receptors and when activated, they release IL-10, CCL2, IL-6 and RANTES [189]. They can differentiate into monocyte-derived macrophages and dendritic cells (DCs) and play an integral part in shaping inflammation and its resolution in tissues, connecting the innate and adaptive system. Intermediate monocytes (CD14 + /CD16 +) express the highest levels of antigen presentation-related molecules (HLA-DR = MHCII) and they secrete TNF, IL-1β, IL-6 and IL-8 [189]. These cytokines are also released by the non-classical monocytes (CD14-/CD16 +), but they do not express MHCII [189], and they have been recently proposed to act as custodians of vasculature by patrolling endothelial cell integrity [7]. Classical monocytes can become intermediate monocytes before finally differentiating into non-classical monocytes in vivo [136]. The analysis of blood monocyte subpopulations in PD has rendered some contradictory results, with some groups reporting no changes [156, 157] while others reported increased classical monocytes with a parallel decrease of the non-classical population [67, 185]. These contradictory results might be due to cohort differences, notably, PD subtype or disease duration might be a crucial factor. Accordingly, our own data show increased classical monocytes in RBD prodromal PD (Farmen, Romero-Ramos et al., unpublished), and in early PD (< 5y), to decrease later in disease (Nissen, Romero-Ramos et al., unpublished). This suggests a response aiming to resolve inflammation early in the disease that later fails as disease advances.

#### Functional changes in monocytes in Parkinson’s disease: abnormal activation, phagocytosis and proliferation

The involvement of monocytes in PD is further corroborated by the suggested inability of the myeloid cells from PD patients to mount a healthy and balanced response to different stimuli, most notably α-syn. We have shown that PD patient PBMCs showed reduced sensitivity to LPS and fibrillar α-syn stimulation and they were unable to modulate the expression of proteins such as CD163, nor efficiently induce cytokine release as compared to control PBMCs [128]. However, studies from the Danzer’s group reported that CD14 + monocytes from PD patients are hyperactive and dysregulated in response to different stimulations such as pathologic α-syn [67, 68]; although, Williams-Gray’s group found no change with CD14 selected cells [184] or with PBMCs [183]. Again, these discrepancies are most likely due to differences in the cohorts, methodology and biomarkers used. Notably, some groups differentiated monocytes to macrophages or pre-selected CD14 + cells for their analysis, while others use PBMCs, which might result in cellular interactions within the culture that would be relevant for the outcome (for example with T-cells). A better consensus is needed in the field of how to approach these functional studies and how these cells respond to α-syn specific stimulation.

Regarding phagocytic capacity of monocytes, two labs reported a decreased functionality in PD patients’ cells [57, 67]. This decrease in phagocytosis seems to be due to α-syn itself, which was found to increase intracellularly [57]. However, another study reported a higher phagocytic capacity in monocytes from PD patients early after diagnosis (< 5y, H and Y 2), suggesting again disease duration as a crucial factor. Notably, the authors report changes only while using autologous serum, *vs.* the standard animal serum, thus highlighting the relevance of the complex disease environment found in vivo, *vs.* the simplification of in vitro assays [184]. Nevertheless, the same group showed recently that this higher phagocytic capacity was not true when α-syn uptake/clearance was specifically assessed (even using autologous serum), thus suggesting a certain selective failure or downregulation in the α-syn uptake process in PD [185]. Remarkably, this occurred despite an increased TLR4 expression in the PD monocytes, that did not lead to a higher response to LPS (a TLR4 activator) or a higher α-syn uptake (as expected based on the suggested TLR4 mediated α-syn clearance [50]). This further suggests a functional impairment of the monocytes in PD patients, which, together with the age-related decrease capacity of macrophages to uptake α-syn, might play an important role in PD progression [12].

Suggesting a strain in immune cells during PD, we reported a reduced survival capacity in vitro of PD-derived PBMCs with a parallel increase in monocytic proliferative capacity, particularly in patients with late age at onset and shorter disease duration [128]. Accordingly, monocyte precursor enrichment is an early event in PD patients [54] that might be associated to the expansion of the classical monocyte population in early disease stages as mentioned before. Altogether, this might indicate an initial compensatory response that aims to resolve the inflammatory event, ultimately lost with time, suggesting that the course of disease progression is a relevant factor to consider when studying immune responses in PD. The in vitro work done so far supports as well a monocytic population that is functionally different than that of healthy subjects. However, better cohort definitions by dividing cases based on their disease duration, subtype or prognosis, is needed to properly define the role of the immune cells.

### Adaptive immunity in Parkinson’s disease: HLA and T cells

Phagocytosis of α-syn will lead the protein to the MHC encoded by the HLA-system (Fig. 1). HLA is a highly polymorphic group of genes subdivided into class I (MHCI) and class II regions (MHCII), both located on chromosome 6. These genes are key for linking innate and adaptive immune responses and are responsible for T cell selection, antigen sampling, activation and induction of adaptive immune responses. Linking genetics to PD risk, GWAS have implicated single nucleotide polymorphisms (SNPs) in HLA-DR which are associated with late onset idiopathic PD, indicating a role for the immune system in PD susceptibility [75]. SNPs in HLA-DR are associated with other autoimmune disorders such as rheumatoid arthritis, multiple sclerosis, and inflammatory bowel disease (IBD). Since the initial GWAS, these results have been replicated implicating multiple MHCII alleles including HLA-DRB5*01 and HLA-DRB1*15:01 [188]. These SNPs reside within a non-coding region of HLA [75, 188] suggesting that they likely affect MHCII expression, which has been confirmed to be higher in PBMCs isolated from SNP-carrying PD patients [94].

In the CNS, MHCII proteins are expressed on antigen presenting cells (APCs) such as CNS resident microglia and border-associated macrophages, and in peripherally infiltrating monocytes and monocyte-derived macrophages. In *postmortem* PD brains, HLA-DR + cells are detected in close proximity to neurons with α-syn pathology [35] in the SN [123] as well as CD4 + and CD8 + T cells surrounding neuromelanin + neurons [16]. Moreover, HLA-DR expression in the CNS correlates with disease severity [89], suggesting that antigen presentation and adaptive immune mechanisms are critical to neurodegeneration. In α-syn viral-vector based animal models of PD, reactive MHCII + microglia [9, 77, 150] as well as infiltrating monocytes/macrophages [78, 79] and T cells have been reported [78, 148, 150, 186]. Genetic deficiency of MHCII [77], the MHCII transcriptional co-activator CIITA [186], and CD4 (Harms et al., unpublished) are neuroprotective indicating a critical role of CNS antigen presentation to CD4 T cells in neurodegeneration. While not implicated in genetic studies, research in *postmortem* tissues have also shown expression of MHCI on neurons in the SN and locus coeruleus of PD patients and are in close proximity to CD8 + T cells [25]. Interestingly, these neurons are IFN-γ responsive and upregulate functional MHCI on the cell surface, actively presenting antigens to CD8 T cells, implicating a novel mechanism of selectively neuronal vulnerability in PD [25].

As mentioned, in *postmortem* PD brains, CD4 + and CD8 + T cells have been detected in the SN [16, 162] near blood vessels and surrounding neuromelanin + neurons [16] suggesting a role for T cells in PD pathogenesis. It is likely that the interaction between the MHCII^+^-APC is important and responsible for elevated T cell derived-cytokine expression, specifically IFN-γ and TNF, in the brain, blood, and CSF observed in PD [13, 187]. In support of T cells driving inflammation in PD, numerous studies over the years have reported changes in T cell subsets, most notably decreases in naïve, CD4 T helper cells (Th), cytotoxic T cells (CD8), and T regulatory (Treg) cells while others have reported no changes or increases in overall numbers (reviewed in [59]). This Th reduction in PD, has been recently associated to a decrease in Th2, Th17 and regulatory T-cell populations; moreover, CD4-Th cells from PD patients show a Th1-biased immune response with increased IFN-γ and TNF production [108]. However, another recent study by Sommer et al. showed increases in Th17 cells in PD relative to controls and follow up studies using these Th17 cells expressing IL-17A from PD patients had direct toxic effects on iPSC-derived neurons expressing IL-17R, suggesting Th17 cells may be regulators of dopaminergic neuronal survival in an experimental model of PD [162]. Overall, these findings suggest an unbalance in Th cells towards the pro-inflammatory phenotypes, which could contribute to the neurodegeneration.

Current research implicates that it may not be necessarily the numbers of particular T cell subsets, but the effector (Teff) response that drives inflammation in PD. Human studies have found not a change in Treg numbers, but a decrease in Treg ability to suppress the activity of Teff cells [153] in vitro, indicating a reduced ability to regulate inflammatory responses in PD. Additionally, we have shown that in vivo T cells react differently to peripheral injections of monomeric and modified α-syn (fibrillar or nitrated), and these conformer responsive T cells work to modulate microglia in the brain [130]. In addition, we also reported that in an α-syn AAV2/5 mouse PD model, Treg cells seem essential in modifying disease phenotypes as vaccination modified Treg populations in the periphery [33], increased the number of Treg cells in the brain and reduced α-syn pathology [148], indicating T cell-modulation as a potential protective strategy.

In support of T cells driving inflammation, a key study by Sulzer and colleagues showed that PBMCs obtained from PD patients were responsive to α-syn peptide fragments [166] supporting the antigenicity of α-syn, a finding that has since been replicated [115]. Their approach showed that α-syn-derived epitopes, particularly epitopes in the pSer129 region (associated with Lewy bodies) are recognized primarily by CD4 + T cells, and also by CD8 + , although less frequently [166]. Interestingly, one particular T cell activating peptide fragment was shown to bind with high affinity to the HLA alleles DRB5*01 and DRB1*15:01, further solidifying the role of α-syn driven antigen presentation and subsequent adaptive immune activation [166]. In a follow up study the authors showed that the α-syn reactivity in T-cells occurs prior to disease diagnosis and is especially high early in the disease and decreases later on, highlighting yet again the relevance of disease stage in the immune response [113]. Interestingly, the α-syn reactive T-cells released IFN-γ and IL-4, associated to Th1 and Th2 responses, respectively. However, they also released IL-10, an anti-inflammatory cytokine, despite not expressing markers of Treg cells [113], which supports an eventual exhaustion of the anti-inflammatory ability as disease progresses. Future studies are essential to determine the dynamic progression of the T cell response, and whether immunotherapeutic T cell targeting strategies are disease modifying in PD.

### Humoral responses: B cells and autoantibodies

While B cells contribute to CNS disease through their actions in the periphery, research into the role of B cells in PD to date have been limited [146]. In steady-state conditions, B cells exist in the CNS parenchyma in low numbers (~ 0.1 cell/cm2) and the perivascular space [5], and this subset of B cells can increase in number and/or effector function [105, 119]. This B cell presence within CNS-associated spaces indicates a role for B cells in immune surveillance and antigen-specific memory and also implicates disease mechanisms that are likely affected by age and neurodegenerative diseases [146]. In PD, B cells have not been detected in *postmortem* brains, however, deposits of IgG have been detected on dopaminergic neurons in the SN and on Lewy bodies in the CNS [131]. In support of age-related phenotypes in PD, it has been shown that autoantibodies decrease in PD, indicating a protective role for B cells by providing the means of extracellular clearance of pathological α-syn [10, 19]. Other studies have found elevated α-syn antibodies in inherited forms of PD [134], or in sporadic PD in the blood [159] and CSF [3, 86]. These contradictory results have been recently reviewed in a meta-analysis, where the authors conclude that differences in cohorts, controls and technical approaches might account for the discrepancies [158]. Although it is yet unclear whether these patient derived anti-α-syn antibodies are neuroprotective or not,  as in vitro assays suggest that antibodies help the α-syn clearance [8], and this is also supported by in vivo studies [47], which led to the current ongoing clinical trials using passive and active immunization.

### Soluble immune biomarkers-predictors of disease outcome?

Due to the accessibility of peripheral biofluids, it has been proposed that immune-related biomarkers could allow for early disease diagnosis and personalized assessment of disease progression. Multiple labs have reported alterations in cytokine and chemokine patterns in PD patient biofluids (Table 2). Two recent meta-analysis reported an increase in several pro- and anti-inflammatory cytokines and other immune related molecules both in CSF and serum of PD patients, suggesting a complex regulation of immune events occurring in parallel in the brain and periphery [102, 141].

**Table 2:** Soluble biomarker changes in PD patients *vs*. healthy controls unless specified (In italics, contradictory results)

Cytokines/Chemokines	Changes in PD	References
TNF, IL-1β, IL-6, IL-2, IL-10, CCL5	↑ blood	[141] (Meta-analysis)
TNF, IL-1β, IL-6, IL-10 IL-1β, IL-6, TGF-β1 IFN- γ	↑ blood ↑ CSF ↓ CSF	[102] (Meta-analysis)
TNF, IL-1β, IL-2,IL-10	↑ blood	[187]
IL-1β, IL-6, IL-2	↑ blood	[101]
IL-2 IL-13, G-CSF	↑ in prefrontal cortex in PD and MSA ↓ in prefrontal cortex in PD and MSA	[145]
IL-1β	↑ blood & PBMCs	[49]
TNF, IL-6, IL-2, CCL2	↑ CSF	[157]
IL-8, CCL2, CCL4 (MIP-1β)	↑ blood of diffuse/malignant PD-LRRK2 *vs.* pure motor phenotype	[17]
IL-8, CCL2, CCL4	↑ blood of GBA-PD	[27]
**Other biomarkers**		
CRP	↑ blood ↑ CSF	[141](Meta-analysis) [102]
NLRP3	↑ blood & brain	[29] [112]
NLRP3,Caspase-1 α-syn Caspase-1, α-syn	↑ PBMCs *↑ blood* *↓ blood*	[49] [49] [185]
β-NGF, DNER	↓ CSF of MSA *vs.* PD patients	[151]
BDNF	↑ blood of diffuse/malignant PD-LRRK2 *vs.* pure motor phenotype	[17]
sCD163	↑ CSF and serum	[127]

Corroborating the inflammation in PD and its detrimental role, increase in the C-reactive protein (CRP), an acute phase protein, can predict cognitive decline [125] PD prognosis [154] and correlated to severe motor symptoms in PD patients [151]. Moreover, the “pro-inflammatory profile” found in the serum of newly diagnosed PD patients (Table 2) was associated with lower motor scores and faster motor decline [187]. Accordingly, the use of anti-TNF is related to lower PD incidence [138] and has also shown a neuroprotective effect in PD models [122]. Another study in patients with early PD showed increased levels of IL-1β, IL-2 and IL-6 in blood (*vs.* controls) [101]. IL-2 is also elevated in the brain of PD patients [145], which is especially relevant, due to its essential role on T-cell survival and activation.

The increase in IL-1β levels in PD patients supports the inflammasome involvement also suggested by the NLRP3 increase in blood [29] and brain of PD patients [112]. Increased NLRP3 protein levels, caspase-1 and IL-1β were seen in PBMCs from PD patients where, once again, plasma levels of IL-1β were increased and correlated with motor severity. α-syn levels in serum were also significantly higher in PD patients and correlated with both motor severity scores and IL-1β expression [49]. In contrast, a recent study reported lower α-syn and caspase-1 levels in PD serum *vs.* controls [185]. Despite the contradictory results, both studies showed a correlation between α-syn and caspase-1 supporting the relation of α-syn in the inflammasome cascade, which requires further research.

Supporting a deleterious role for the recruitment of immune cells, several studies suggest that chemokines are especially relevant for PD progression and in more aggressive PD forms. Accordingly, in a longitudinal study, CCL3 (MIP1α) and CCL2 were the serum biomarkers contributing the most to the predictive models of motor severity [2]. And indeed, in the CSF of PD patients, an increase in activated T cells and non-classical monocytes has been observed, together with elevated levels of pro-inflammatory cytokines and CCL2 [157]. Furthermore, chemokines IL-8, CCL2 and CCL4 have been shown to be especially relevant in aggressive PD subtypes such in PD-LRRK2 patients with diffuse/malignant PD [17], and in GBA-PD, where IL-8 was correlated with higher cognitive deficits [27]. In fact, monocyte activation seems to be especially relevant in the cognitive component of PD. Accordingly PD patients with higher risk to develop cognitive symptoms, showed more significant changes in the monocytic population [185]. Moreover, we have shown that soluble CD163, which is exclusively produced by monocytes/macrophages during activation, is increased in PD CSF and correlates directly with α-syn and indirectly with cognitive scores [127]. Thus, higher monocytic activation was associated to worse cognition. Furthermore, in PD with dementia, levels of immune activation, assessed by PK11195 PET were significantly correlated with decrease cognitive scores [46]. Therefore, the immune component seems more readily manifested in cases where the course of PD progression is aggressive and associated with worse cognitive impairment.

### Vagus nerve, gut, and peripheral inflammation

The Braak hypothesis suggests that α-syn-pathology and PD might start in the periphery within the gastrointestinal track, and through the vagus nerve and dorsal motor nucleus (DMN) progress towards the brain; consequently, pathology is seen in the peripheral nervous system (PNS) and CNS. Indeed, peripheral denervation associated with the vagus nerve has been shown in PD (reviewed in [15]). This will have a direct repercussion in the immune system, specifically through the so called inflammatory reflex: a bidirectional anti-inflammatory brain-periphery communication which relays in the DMV and acetylcholine signaling. This involves spleen, gut, T-cells, macrophages and several neuronal nuclei (reviewed in [30]). Borghammer et al., has recently proposed a new hypothesis of two PD subtypes based on whether the patients showed the first signs of neurodegeneration: in the PNS, body-first-PD, or in the CNS: *brain-first-*PD [85]. Accordingly, in rodent α-syn based models, α-syn pathology can spread bidirectionally between gut-brain [174, 175]. While the proposed *body-first* shows RBD signs, fast progression and more cognitive impairment, the *brain-first* are RBD-negative and show a milder disease progression. In light of Borghammer’s theory, the first myeloid cell to encounter aggregated α-syn would be peripheral monocytes and macrophages in the *body-first* PD type, *vs.* microglia in the *brain-first*-PD subtype. Moreover, the loss of immune control exerted by the DMN will occur early in the *body-first*-PD, which might contribute to the faster and more severe progression of this subtype. Accordingly, RBD patients (putative *body-first*-PD) show a decrease in the anti-inflammatory cytokine IL-10, rather than a pro-inflammatory profile, that might be related to the DMN affection [100].

As a direct consequence of the findings of the PNS degeneration in PD, studies regarding gut-brain axis and microbiota influence in neurodegeneration have been of interest. Interestingly, in the rAAV- α-syn rodent model, overexpression of α-syn in the SN led to enteric nervous system changes and altered microbiota [129], while in transgenic α-syn PD model the microbiota influenced the neurodegeneration process [147]. Indeed, changes in microbiota have been related to PD [167], but also to RBD, suggesting that this might be a factor of very early relevance [81]. The influence of microbiota on shaping the immune system has long been known, but this is a novel exciting concept within the PD field (reviewed in [88]). The relevance of the gastrointestinal track has also been investigated in epidemiological studies suggesting that the risk and incidence of PD is lower in those persons that underwent vagotomy or appendectomy earlier in life [168, 169]. Within this context, inflammatory events in the digestive tube (like in the appendix) seem of high relevance, particularly due to the enrichment of α-syn of the myenteric plexus of the appendix and the presence of macrophages with engulfed α-syn in the area [66]. This is also supported by the relation of PD with IBD [18]. Inflammation and dysbiosis, will result into a leaky gut wall that may cause immune activation that promotes neurodegeneration (Fig. 1).

A recent study found increased levels of endotoxin in PD patients’ blood, especially those with a higher risk for dementia, suggesting an active role for bacterial infection in the outcome of the disease [185]. This is in agreement with the synergistic neurotoxic effect of chronic (peripheral) LPS and α-syn shown by Hong’s lab [56, 193]. Interestingly, a study in WT mice showed that intraperitoneal LPS injection, prior to α-syn peripheral intravenous administration, led to α-syn internalization by inflammatory monocytes that in turn can infiltrate the brain, suggesting that the peripheral activated monocytes can act as a Trojan horse in PD, promoting the entrance of peripheral (modified) α-syn into the CNS [137]. Altogether this has contributed to the double-hit hypothesis of PD and further corroborate the complex and multisystem nature of PD (see [92]).

### Other factors related to α-synuclein and the immune response in Parkinson’s disease

Lysosomal dysfunction seems to be at the center of α-syn pathology, a process of special relevance in glia (see review [52]); in that regard we would shortly discuss two proteins genetically related to PD: LRRK2 and glucocerebrosidase (Gcase). LRRK2 is expressed in immune cells, but considerably higher in monocytes and microglia than in T cells [58], suggesting LRRK2 as a key player in innate immunity. Genomic studies implicate LRRK2 mutations not only in PD, but also in other inflammatory disorders, specifically IBD, further supporting the functional role of LRRK2 in immune cells [179]. Within the cell, LRRK2 has been implicated in phagocytosis through the autophagy/lysosomal degradation pathway [155], and LRKK2 mutation leads to abnormal chaperone mediated autophagy and α-syn accumulation [82]. Rab proteins have been identified as substrates for LRRK2 kinase activity [114, 143], implicating a role for LRRK2 in membrane trafficking and regulation of immune cell function such as phagocytosis, exocytosis, and antigen presentation. LRRK2 is also implicated in modulation of cell-surface markers in monocytes and microglia [172] and regulation of cytokine production [124], and pathogenic mutations are associated with enhanced neuroinflammation and neurodegeneration upon systemic inflammation [106].

As mentioned, immune cells will respond to α-syn promoting inflammation and protein clearance/degradation. These two processes might be mediated by LRRK2, due to common receptor pathways (to both α-syn and LRRK2), or by LRRK2- mediated direction to autophagy degradation [37]. Pathogenic mutations of LRRK2 seem to compromise microglia ability to internalize and degrade α-syn [99]. In addition, LRRK2 expression and phosphorylation increase in microglia and monocytes upon TLR2 or TLR4 stimulation [124, 155], both α-syn interactors. Although LRRK2 response was slightly different in monocytes *vs.* microglia cell lines, both showed autophagic deficits upon LRRK2 *knock-down* [155], further implicating LRRK2 in the regulation of lysosomal degradation in myeloid cells. In a human study, both asymptomatic and PD patient carriers of LRRK2 G2019S mutation showed increased levels of peripheral inflammatory cytokines [43], suggesting a pathological contribution of LRRK2 mutations in mediating peripheral immune response. Altogether, this suggests that dysregulation of these and other LRRK2-associated signaling pathways might relate to α-syn accumulation and consequent neuroinflammation. For relevant additional reading on the role of LRRK2 in immune system modulation we refer the reader to [22, 179].

Mutations in the GBA1 gene, encoding the lysosomal enzyme Gcase are responsible for causing the autosomal lipid storage disorder, Gaucher disease (GD), characterized by the deposition of glucocerebrosides in monocytes-macrophages. GBA1 mutations are the most important genetic risk factors for PD [161]. *Postmortem* analysis of brain tissue from patients with GBA-PD showed increased levels of α-syn in the SN and a significant correlation between the reduction in the Gcase protein levels and an increase in p129/total α-syn [69]. Indeed, a biochemical connection between GBA and α-syn has been reported, with GBA mutations leading to accumulation of α-syn in human cells [36] and α-synuclein pathology in mouse models, associated to autophagy failure [152]. Additionally, α-syn pathology itself can lead to lysosomal dysfunction [121]. Thus, the additive effect of GBA mutations into the lysosomal failure and lipid accumulation might explain the higher PD risk observed. Remarkably, GBA carriers without manifested PD show immune activation by PK1195 PET in SN [126], supporting an early role for the immune system.

Interestingly LRRK2 and GCase seem to converge since LRRK2 mutation led to decreased GCase in patient-derived cells, and inhibition of LRRK2 kinase activity increased GCase activity in neurons with either *LRRK2* or *GBA1* mutations.[191]. Accordingly, activation of GCase can rescue neuronal health in iPSC models of genetic GBA and LRRK2 PD [20]. Gcase was also protective in a model of peripheral synucleinopathy based on injections of α-syn PFF in the gut, supporting the importance of integrity of lysosomal function in the peripheral pathology in PD [28]. Accordingly, Gcase activity was found reduced in monocytes from idiopathic PD patients [6] further supporting lysosomal dysfunction in peripheral myeloid cells. In monocyte-derived macrophages from patients with type 1 GD, inflammasome activation showed to be the result of impaired lysosomal autophagy [1]. In these cells, the increase in p62 led to activation of p65-NFκB pathway, which per se promoted the expression of inflammatory cytokines and increased IL-1β secretion [1], providing a link between inflammation, lysosome storage and autophagy impairment, three major processes with possible relevant implications for α-syn clearance and the PD pathogenesis. More work is needed to elucidate the relevance such mutations in immune cells in the α-syn neurodegenerative process. For further reading see: [126, 163].

## Conclusions

It is increasingly clear that the immune system is a relevant component of the disease pathogenesis in PD, as there is strong evidence for innate and adaptive immune mechanisms in both human disease and α-syn based animal models. As research progresses and methodologies for detection evolve, it is  evident that these changes in the immune component in PD occur early and change dynamically with disease progression. Although previously thought to involve CNS-specific immune mechanisms, research has now shown that both brain, as well as peripheral immune cells, are involved in this inflammatory event providing strong evidence for innate and adaptive immune system crosstalk in the CNS and periphery.

At the core of disease pathogenesis, α-syn has proven to be a key player as it not only contributes to the hallmark pathology observed in PD *postmortem* tissue, but is also key in activating and driving inflammation and neurodegeneration in human PD. These central pathways of α-syn-driven innate and adaptive immune activation have been recently dissected in α-syn-based animal models, which have been instrumental in modelling human disease. Using these animal models in parallel with human-based studies allowed to identify novel pathways potentially driving neurodegeneration in the PNS and CNS that involve not only the immune system, but also implicate the gut microbiome, genetic predisposition, and environmental immune challenges. We believe that analysis of longitudinal changes in the inflammatory profile in patients, in combination with peripheral immune profiling, gut microbiome testing, α-syn blood and CSF analysis, and PET imaging may provide a unique opportunity for discovery or detection of unique immune based biomarkers to predict disease outcomes and progression. Early detection and a clear understanding of the progressive immune system involvement in PD may lead to novel therapeutics that not only target CNS-specific  components, but also target the periphery, offering neuroprotection and halting disease progression.
